# Comparison of sensitivity and specificity of three diagnostic tests to detect *Schistosoma mansoni* infections in school children in Mwanza region, Tanzania

**DOI:** 10.1371/journal.pone.0202499

**Published:** 2018-08-22

**Authors:** Antje Fuss, Humphrey Deogratias Mazigo, Dennis Tappe, Christa Kasang, Andreas Mueller

**Affiliations:** 1 Medical Mission Institute, Wuerzburg, Germany; 2 Catholic University of Health and Allied Sciences, Mwanza, Tanzania; 3 Bernhard Nocht Institute for Tropical Medicine, Hamburg, Germany; 4 Klinikum Wuerzburg Mitte gGmbH, Medical Mission Hospital, Dept. of Tropical Medicine, Wuerzburg, Germany; Centers for Disease Control and Prevention, UNITED STATES

## Abstract

**Background:**

Schistosomiasis remains one of the most prevalent parasitic infections in the world and has significant economic and public health consequences, particularly in poor communities. Reliable and accurate diagnosis plays a key role in surveillance, prevention and control of schistosomiasis. Currently, the microscopic Kato Katz (KK) stool thick smear technique is the most commonly used method to diagnose *Schistosoma mansoni* infections in epidemiological surveys. It is well-known that the sensitivity of this parasitological method decreases when infection intensities are moderate to low, however. The urine-based Point-of Care Circulating Cathodic Antigen (POC-CCA) test has been extensively evaluated as a further diagnostic tool. Several studies have shown that the POC-CCA test is more sensitive but less specific than the KK method. However, to clarify the meaning of inconsistent results between KK and POC-CCA tests in clinical routine, this study compares the accuracy of microscopy and POC-CCA versus real-time polymerase chain reaction (real-time PCR) results of urine and faecal samples from African school children participants.

**Methodology:**

This was a school-based cross-sectional study conducted in 2015 among 305 school children aged 7–16 years from two primary schools located in Ilemela and Magu Districts, north-western Tanzania. Single stool and urine samples were collected from each participant and examined for the presence of *Schistosoma mansoni* eggs, parasite antigen, and parasite DNA using KK thick smears, POC-CCA tests, and real-time PCR, respectively.

**Principal findings:**

The prevalence of *S*. *mansoni* infection, calculated by KK was 85.2%, by real-time PCR 92.9% and by POC-CCA 94.9%. In comparison to KK, the POC-CCA and real-time PCR tests had sensitivities of 89.7% and 99.5% and specificities of 22.73% and 29.55%, respectively. However, due to the known limitations of the KK assay, we also used latent class analysis (LCA) that included POC-CCA, KK, and schistosome-specific real-time PCR results to determine their sensitivities and specificities. The POC-CCA test had the highest sensitivity (99.5%) and a specificity of 63.4% by LCA and the real-time PCR test had a sensitivity of 98.7% and the highest specificity (81.2%).

**Conclusion:**

In moderate and high prevalence areas, the POC-CCA cassette test is more sensitive than the KK method and can be used for screening and geographical mapping of *S*. *mansoni* infections. Real-time PCR is highly sensitive and also shows the highest specificity among the 3 investigated diagnostic procedures. It can offer added value in diagnosing schistosomiasis.

## Introduction

Schistosomiasis is one of the poverty-related neglected tropical diseases that affects more than 230 million people worldwide [1]. More than 90% of the estimated cases of schistosomiasis occur in sub-Saharan Africa (SSA), with the largest numbers in Nigeria followed by the United Republic of Tanzania [2,3]. In SSA, schistosomiasis is caused mainly by two species, *Schistosoma mansoni* and *Schistosoma haematobium* [4,5]. Data from several studies show that the prevalence of schistosomiasis in Tanzania varies from one region to another, with a prevalence up to 80% in some areas [4,6,7]. Especially populations living around Lake Victoria have a high risk of infection [6–8], where snails of the *Biomphalaria* genus occur which are the obligatory intermediate hosts for *S*. *mansoni* [9]. The prevalence, infection intensity and transmission intensity is determined by various causes such as human behaviour, ecology and biological factors related to the parasite [4]. According to a research study by Colley *et al*. (2014), in endemic areas the initial infection often occurs as early as in children aged 2 to 3 years [1]. The burden of infection increases during the next 10 years of life because the children are repeatedly re-infected [1]. The highest prevalence and intensity of infection are commonly described in young adolescents [1,3].

For geographical mapping and field-based control of schistosomiasis, microscopic examination of schistosome eggs in faecal or urine samples is recommended by the World Health Organization (WHO) [1,10]. Due to their typical size and shape, the egg of *S*. *mansoni* and *S*. *haematobium* can be easily detected and identified under the light microscope using the Kato Katz (KK) technique or urine filtration, respectively. The KK technique is 100% specific [11], but its sensitivity in detecting the infection in individuals with light infection intensity is very low [5]. It is important to know that such cases can still infect snails (vector) and keep the transmission ongoing. In addition, parasitological methods are not sufficient for diagnosing current infections in which worms have not yet started laying eggs. To improve the sensitivity, collection of at least three stool samples on three consecutive days is needed in some areas, especially in regions with low infection intensity or post treatment [5,12–14]. Another commonly used method to diagnose schistosomiasis is the Point-of Care Circulating Cathodic Antigen (POC-CCA) urine test [15]. The Circulating Cathodic Antigen (CCA) is released with the vomitus of feeding adult schistosomes into the blood and secreted in urine [16]. The CCA provides a useful marker of the worm burden [15]. The major advantages of the POC-CCA test cassette includes its easy handling under field conditions and minimal practical training requirements for its application [11]. This lateral flow cassette assay has been tested in numerous studies and seems to be more sensitive than the KK technique [17,18]. Moreover, the intensity of the positive test band allows an estimation of infection intensity in infected individuals [11,19]. Nevertheless, the specificity of the POC-CCA is influenced by cross-reaction with other helminth infections and most importantly may produce false-positives especially in moderate and low prevalence areas [13]. This may lead to an over-estimation of prevalence [11]. Neither KK nor the POC-CCA test can detect all cases and a more sensitive and specific test is needed for diagnostic purposes and treatment control. A method with very high sensitivity and specificity is represented by the detection of *S*. *mansoni* DNA in faeces, sera, plasma or urine using the polymerase chain reaction (PCR) [13,20,21]. Various target genes have been tested and established for molecular detection of *S*. *mansoni* [22–27]. A frequently used sequence is SM1-7 [23,24,28,29]. SM1-7 is a highly repeated DNA sequence from *S*. *mansoni* which contains 121 bp tandem repeats (600,000 copies per cell) and comprises at least 12% of the *S*. *mansoni* genome of both sexes [22]. These DNA fragments can be detected by PCR and show no cross-reactivity to other helminths [23]. However, the sensitivity of PCR is greatly influenced by the quality of the extracted DNA templates [30].

Due to numerous mass drug campaigns and implemented elimination programs the conditions of schistosomiasis in endemic communities has changed. In the current situation, it is imperative to establish and evaluate highly sensitive detection methods, to diagnose also light infections and to verify successful treatment. On the one hand, the KK- technique fails to detect all cases of schistosome infection. On the other hand, the use of the POC-CCA test cassette may lead to an overestimation of the true prevalence because of false positives. These results emphasize the urgent need to further compare and understand the variability between POC-CCA and KK results with highly sensitive and specific methods. Although many studies have been carried out to evaluate the POC-CCA test and KK simultaneously, only few surveys have attempted to include molecular methods [13,18,31].

Here, we present the results of a study that compares the diagnostic accuracy of microscopy (KK method) and POC-CCA versus real-time PCR in urine and faecal samples of school children from an area in Tanzania that is highly endemic for *S*. *mansoni* infection. In the absence of a good reference method, sensitivity and specificity were determined empirically and by means of latent class analysis (LCA).

## Materials and methods

### Study area and population

A prospective school-based cross-sectional study was conducted in March 2015 among 305 school children aged between 7 to 16 years attending Kayenze and Chabula primary schools located in the Ilemela and Magu districts at the southern shore of Lake Victoria, north-western Tanzania.

### Stool collection and microscopic examination

A single stool sample was collected from all study participants and analysed by real-time PCR and KK thick smear technique. For the KK method two thick smears were prepared from different parts of the same stool sample using a template of 41.7 mg (Vestergaard Frandsen, Lausanne, Switzerland), following a standard protocol [10,32]. After 24 hours, the smears were independently examined for *S*. *mansoni* eggs by two experienced laboratory technicians of the National Institute for Medical Research (NIMR) laboratory. For quality assurance, 10% of the negative and positive KK thick smears were re-examined by a third laboratory technician. In addition, faecal samples were preserved in 70% ethanol at ambient temperature for real-time PCR analyses 2 weeks later.

### Urine specimen collection and testing

Urine specimens were collected from each of the participating children, who had also provided a stool sample. The urine POC-CCA cassette test was blindly performed according to the protocol and procedures described by the manufacturer (Rapid Diagnostics, Pretoria, South Africa). POC-CCA test results were scored on four-point scale: negative, positive 1 (trace readings included), positive 2, and positive 3. For rapid detection of *S*. *haematobium* infections, we used the two-step approach recommended by Robinson *et al*. whereby haematuria-positive urine samples are subsequently examined by urine filtration [33]. Therefore, each urine sample was examined with reagent strips (Roche Combur 9) for microhaematuria. For this purpose, reagent strips were immersed in urine and removed immediately. The strip-results were read after 1–2 minutes by direct comparison with the colour scale provided with the test. Samples with microhaematuria were subjected to a filtration method for detection of *S*. *haematobium* eggs. A volume of 20 ml urine was filtered through a syringe filter with 20 μm mesh size (Millipore Art. No. NY2002500), and filters were examined by microscopy for the presence of *S*. *haematobium* eggs [33–35].

### DNA isolation

For isolating DNA, the ethanol-fixed faecal suspension was centrifuged at 9,500 g for 1 min. The pellet was washed twice with PBS. DNA was isolated using the QIAamp DNA Stool Mini Kit (Qiagen, Hilden, Germany). Extracted DNA was eluted in 200 μl buffer AE and a 2.5 μl aliquot was tested by real-time PCR.

### Amplification by real-time PCR

*Schistosoma mansoni* DNA samples were amplified and detected using a set of primers and probes complementary to a 121bp tandem repeat sequence of *S*. *mansoni* strain SM 1–7 (GenBank accession number M61098) described by Hamburger *et al*. [22]. Primer sequences were: Sm FW 5′-CCG ACC AAC CGT TCT ATG A-3′; Sm RV 5′CAC GC TCT CGC AAA TAA TCT AAA-3′ (19); Sm probe 5′-[FAM] TCG TTG TAT CTC CGA AAC CAC TGG ACG [(BHQ1])-3′ all synthesized by Eurofins Genomics, Ebersberg, Germany.

The 25 μl reaction mix contained 2.5 μl DNA, 1x QuantiFast Pathogen Master Mix (QuantiFast ® Pathogen PCR + IC Kit, Qiagen, Hilden, Germany), 400 nM of each Sm Primer and 200 nM of Sm probe. The PCR runs consisted of an initial step of 5 min at 95°C followed by 40 successive cycles of 15 s at 95°C and 30 s at 60°C. The reaction was run on the StepOne real-time PCR system (Applied Biosystems). DNA detection was expressed by cycle threshold (Ct)—values. In every run, the non-template control was negative (Ct = 0), the positive control was positive and the internal control to test successful amplification and to exclude the presence of PCR inhibitors was positive (Ct < 33). A test was considered positive when the threshold was attained within 38 PCR cycles (Ct < 38).

### Ethical consideration

Written informed consent was obtained from all parents or legal guardians of the school children. This project was reviewed and specifically approved by the Catholic University of Health and Allied Sciences (CUHAS), Ethics Review Board, Mwanza, Tanzania (Research Clearance Certificate No CREC/062/2014). Participants who were positive by microscopic examination or POC-CCA test cassette for *S*. *mansoni* or S. *haematobium* were treated with praziquantel using the WHO recommended clinical dosage (40 mg/kg body weight).

### Statistical analyses

Collected data were entered in a Microsoft Excel spreadsheet, checked and validated. Only samples with data from three diagnostic tests were considered for analysis. Results were recorded for all tests and converted to numerical values (1 = positive and 0 = negative) for analysis. Tables of prevalence and intensity of infection were generated (Tables 1 and 2). Disease prevalence was considered based on the number of positive cases by each diagnostic test. The arithmetic mean egg count was calculated as the average egg count of the two KK smears, and classes of intensity of *S*. *mansoni* were determined as light (1–99 mean EPG per gram (EPG), moderate (100–399 EPG) and heavy (> 400 EPG). The thresholds are set according to the values published by the WHO [36]. In addition, intensity of infection was determined by the Ct value of the real-time PCR (reflecting faecal parasite-specific DNA loads; low intensity infection Ct > 30, high intensity infection Ct < 30) and the intensity of the POC-CCA cassette test band (light = 1+, moderate = 2+ and heavy = 3+). The relationship between the intensity of infection determined by KK, POC-CCA and Ct values of real-time PCR results were examined by the Spearman correlation test. A p-value lower than 0.05 was considered statistically significant. Fisher's exact test was used to look for significant differences in the sensitivity of POC-CCA urine rapid test for the diagnosis of *S*. *mansoni* infections with low, moderate or high intensities. Bonferroni corrected p values were used.

**Table 1:** Prevalence of *S*. *mansoni* according to each diagnostic method.

Test Method	n tested	n positive	% positive (CI*)
Kato Katz	297	253	85.2 (81.2, 89.2)
POC-CCA	297	282	94.9 (92.4, 97.4)
Real-time PCR	297	276	92.9 (90.0, 95.8)

* Exact 95% confidence interval.

**Table 2:** Intensity of infection categories for *S*. *mansoni* by each examined diagnostic test.

Diagnostic test and intensity category	297 tested n (%)
**Kato Katz**	
Negative	44 (14.8)
Light (1–99 EPG)	91 (30.6)
Medium (100–399 EPG)	116 (39.1)
Heavy (≥ 400 EPG)	46 (15.5)
**POC-CCA**	
Negative	15 (5.1)
Light (+)	59 (19.9)
Medium (++)	115 (38.7)
Heavy (+++)	108 (36.4)
**Real-time PCR**	
Negative (Ct > 38)	21 (7.1)
Light (30 > Ct ≤ 38)	56 (18.9)
Heavy (Ct ≤ 30)	220 (74.1)

Sensitivities and specificities were first estimated using Kato Katz method as the reference test. The calculation was done using MedCalc 17.9.6 (MedCalc Software, Belgium). For percentage values, 95% confidence intervals (95% CI) were estimated using the exact method. Because we know that the KK method is not a perfect reference test, LCA was also performed to calculate the sensitivity and specificity [37,38]. To calculate these parameters, the poLCA package for R was used [36,39]. The results of POC-CCA, microscopy and real-time PCR tests were combined as indicators of an underlying latent class. In the current study, we consider the true *S*. *mansoni* infection status to be a latent variable with two categories: ‘infected’ and ‘non-infected’. Sensitivity and specificity were estimated for each test by relating the true disease class and the observed test results.

These analyses were carried out using IBM SPSS Statistics version 24 (SPSS Inc., Chicago, USA) and software R version 3.5.0 (The R Foundation for statistical Computing, Vienna, Austria).

## Results

### Demographic information of study participants

A total of 305 school children were enrolled in the two primary schools. Complete parasitological data (duplicate KK, POC-CCA cassette tests and real-time PCR) were available for 297 children (97.4%), 98 (33%) from Chabula and 199 (67%) from Kayenze primary schools. There were 146 girls (49.2%) and 151 boys with a mean age of 10.45 years (range: 7 to 16 years).

### Prevalence and intensity of *Schistosoma mansoni* infection

Prevalence of *S*. *mansoni* infection was 85.2% (95% CI 81.2–89.2) by KK, 92.9% (95% CI 90.0–95.8) by real-time PCR and 94.9% (95% CI 92.4–97.4) by POC-CCA (Table 1). Overall, there were 12 haematuria-positive urine specimens, which were subsequently examined by urine filtration. In samples of only 2 children eggs of *S*. *haematobium* were detected, one of the two children had a co-infection with *S*. *haematobium* and *S*. *mansoni*.

Of 253 children found positive for *S*. *mansoni* using two KK thick smears, 91 (36%) had light, 116 (45.8%) moderate and 46 (18.2%) heavy *S*. *mansoni* infection intensities (EPG = 266.47 ± 312.96).

Of 282 children found positive for *S*. *mansoni* using the POC-CCA cassette test, 108 showed a strong intensity of the POC-CCA cassette test band colour, while 115 showed a moderate and 59 a weak intensity. By using real-time PCR, 276 children were found positive for *S*. *mansoni*. Of those, 56 children had a weak intensity of infection (Ct > 30) and 220 showed a heavy intensity of infection (Ct ≤ 30) (Table 2).

Of the 297 tested samples, 243 (81.8%) were *S*. *mansoni* positive and 8 (2.7%) were *S*. *mansoni* negative by all three test methods. Among 44 (14.8%) microscopically negative samples, 29 were *S*. *mansoni* positive in both POC-CCA and real-time PCR. In 7 *S*. *mansoni* egg positive samples, only one of the other two tests was also positive. In 3.4% (10) of the samples *S*. *mansoni* infection was detectable only by one test (3 samples by KK, 2 samples by real-time PCR, 5 samples by POC-CCA).

### Sensitivities and specificities of the diagnostic assays

In order to determine the diagnostic parameters of the used tests, on the one hand the KK test was used as reference method (despite its known limitations) and on the other hand LCA was performed (Table 3).

**Table 3:** Sensitivities and specificities of tests in comparison to Kato Katz and LCA.

**Kato Katz as Gold Standard**		
**Test Method**	Sensitivity, % (CI*)	Specificity, % (CI*)
Kato Katz	N/A	N/A
POC-CCA	98.27 (96.8, 99.8)	22.73 (18.0, 27.5)
Real-time PCR	96.84 (94.9, 98.8)	29.55 (24.4, 34.7)
**Latent Class Analysis**		
**Test Method**	Sensitivity, % (CI*)	Specificity, % (CI*)
Kato Katz	89.7 (85.9, 93.5)	72.8 (46.3, 99.4)
POC-CCA	99.5 (98.1, 100)	63.4 (34.6, 92.1)
Real-time PCR	98.7 (96.5, 100)	81.2 (54.7, 100)

* Exact 95% confidence interval.

The sensitivity of the POC-CCA test (98.3%, 95% CI: 96.8–99.8%) and the real-time PCR method (96.8%, 95% CI: 94.9–98.8%) was high when KK was used as a reference method. However, the specificity of both tests was very low, 22.7% for POC-CCA test and 29.6% for real-time PCR assay.

In addition, we performed LCA to determine the infection status of the participants and to calculate the diagnostic parameters of the three test methods used. The sensitivity of real-time PCR (98.7%) and POC-CCA (99.5%) was also very high, the sensitivity of the KK method was the lowest (89.7%). The specificities calculated with LCA were considerably higher than the values calculated with KK as reference. The highest specificity was demonstrated by the real-time PCR assay (81.2%), followed by the KK method (72.8%). The lowest specificity was seen in the POC-CCA test (63.4%).

### Correlation of Ct values, EPG and POC-CCA test band intensities

**Fig 1:**
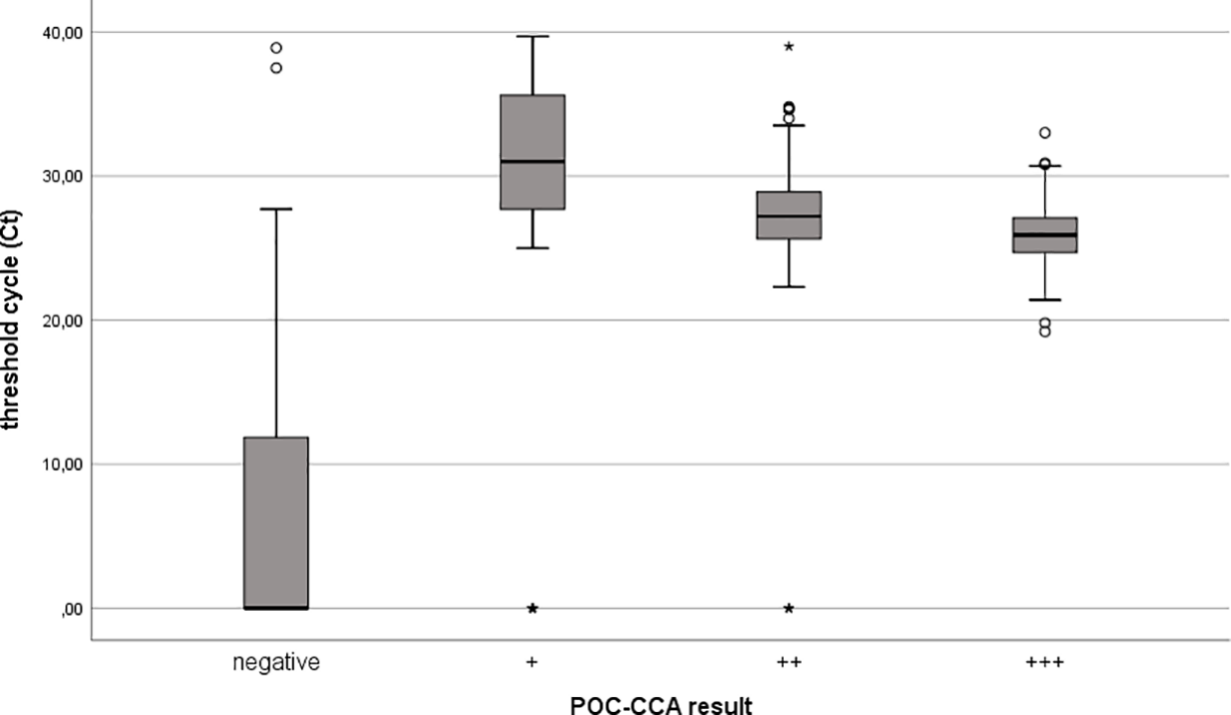
shows a negative correlation (Spearman's correlation coefficient = -0.28; p < 0.001) between the intensity of *S*. *mansoni* infection determined by POC-CCA test (colour scores) and threshold cycle (Ct) values of *S*. *mansoni* specific real-time PCR.

**Fig 2:**
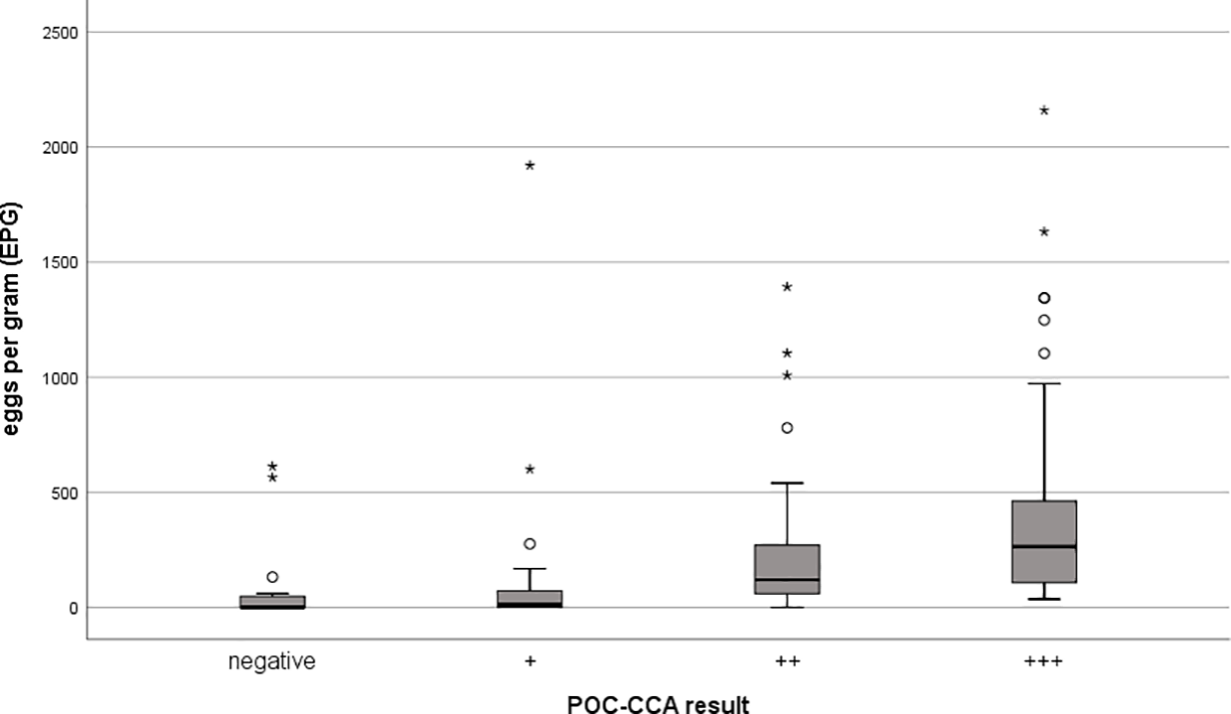
shows that the number of eggs per gram (EPG) of the KK test correlates positively with the intensity of POC-CCA test band (Spearman's correlation coefficient = 0.57; p < 0.001).

**Fig 3:**
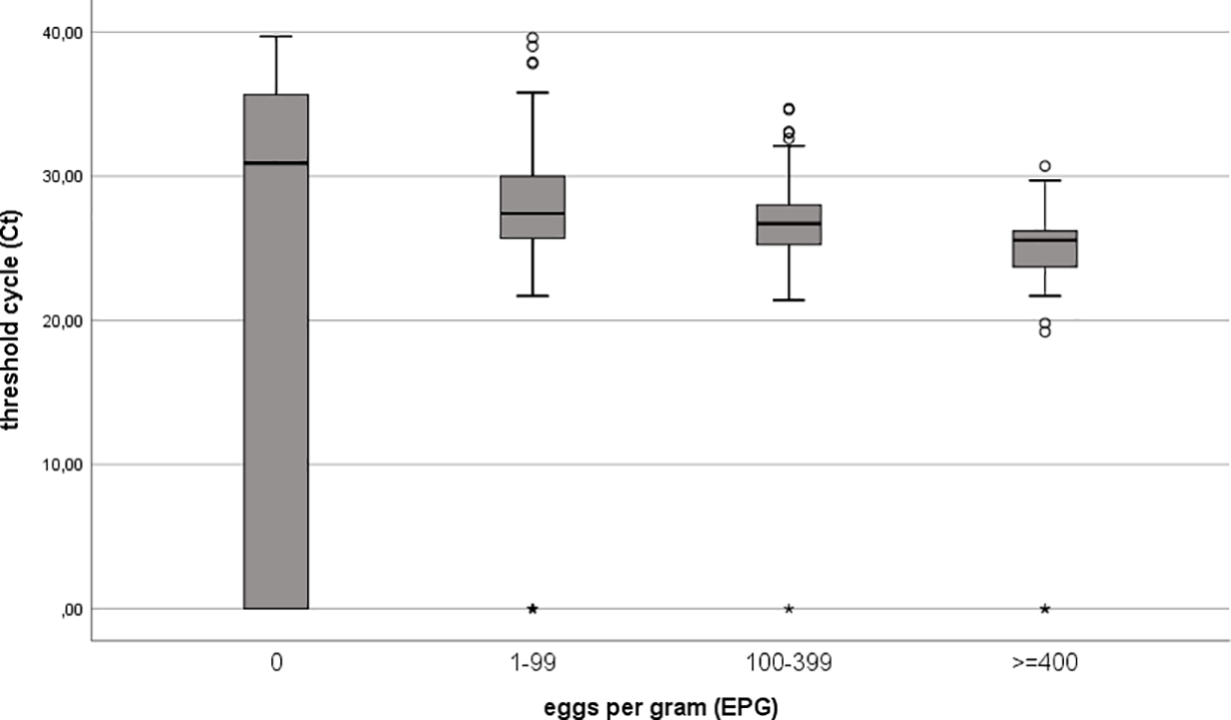
Threshold cycle (Ct) values of *S*. *mansoni* specific real-time PCR correlate negatively with the level of schistosome infection (determined by the Kato Katz method, expressed in eggs per gram (EPG)) (Spearman's correlation coefficient = -0.32; p < 0.001). *S*. *mansoni* real-time PCR-positive samples show a decreasing Ct value with increasing egg count category.

We compared Ct values of real-time PCR and EPG of KK test with the POC-CCA test band strength to show that both are related to the intensity of the POC-CCA (Figs 1 and 2). A positive association between increasing intensity of the POC-CCA test band and faecal egg count was observed (r = 0.57). A negative association between increasing intensity of the POC-CCA test band and Ct values of the real-time PCR method was seen (r = -0.28). In addition, we were able to show that there was a negative correlation between the Ct value of real-time PCR and the number of EPG of stool (r = -0.32) (Fig 3).

**Fig 4:**
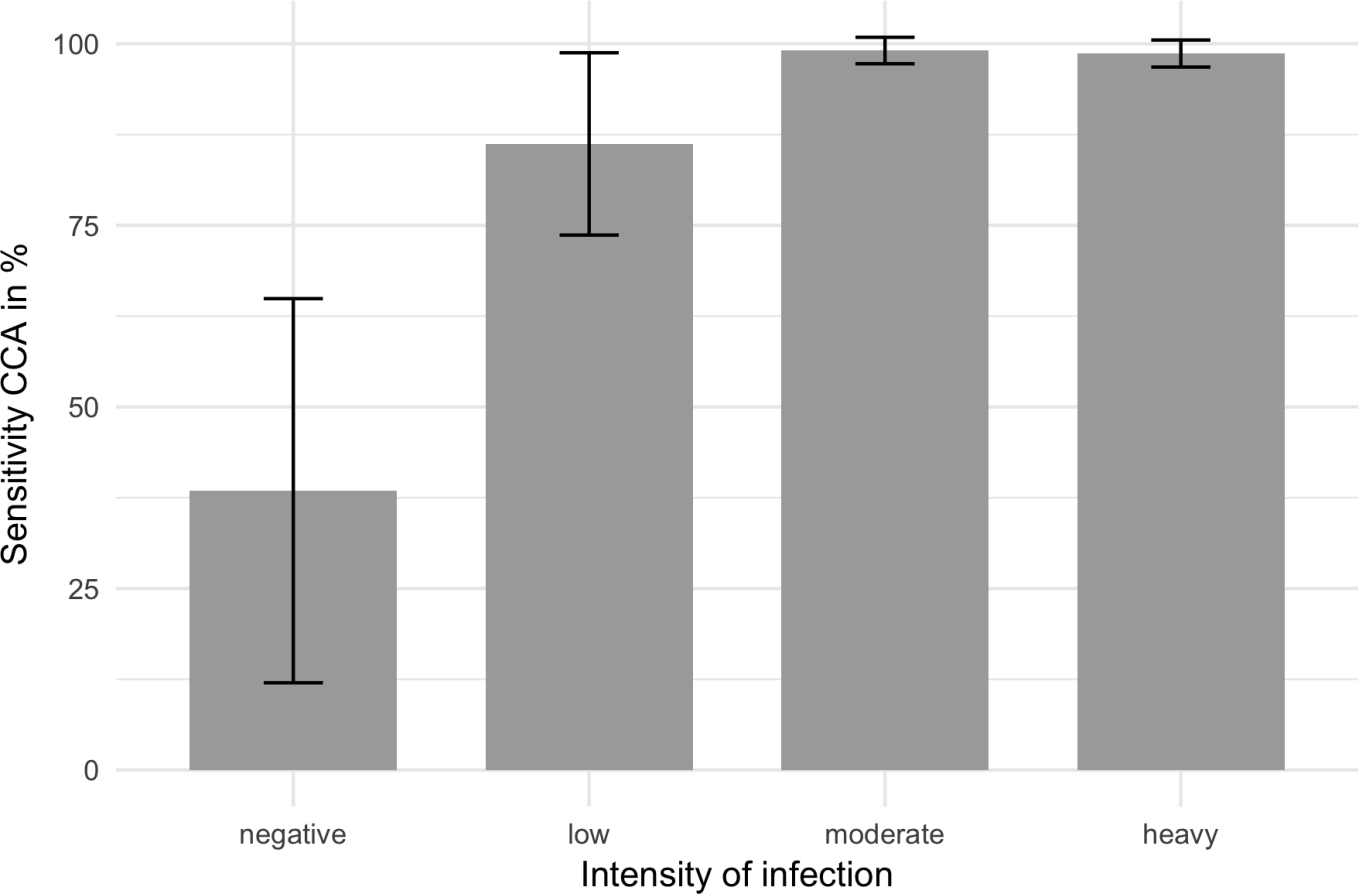
Sensitivity of *Schistosoma* POC-CCA urine rapid test and 95% intervals for the diagnosis of *S*. *mansoni* infections with low, moderate or high intensities determined by egg counts in stool or threshold cycle (Ct) values of real-time PCR results. Low intensity infection: KK 1–99 eggs per gram faeces (EPG) or Ct-value of real-time PCR > 30. Moderate intensity infections: KK 100–399 EPG or Ct-value < 30. High intensity infection: KK > 400 EPG and Ct-value of real-time PCR < 30. The specificity of POC-CCA test amounts to 61.5%.

To determine if POC-CCA results were affected by the intensities of *S*. *mansoni* infections, the data were analysed considering the different intensity classes estimated from two KK readings and Ct-values of real-time PCR. The results are shown in Fig 4. Calculations done with Fisher`s exact test showed that the sensitivity of low-intensity infections deviates significantly from moderate or severe infection intensities (corrected p-value = 0.042). In moderate and heavily infected children the sensitivity of POC-CCA test was 99.1% and 98.6%, respectively. The sensitivity of POC-CCA test decreases in low infection settings (86.2%). Of the 13 children negative for KK and real-time PCR, 5 were positive in POC-CCA test (38.5%).

## Discussion

An accurate and convenient method is essential to ensure a reliable diagnosis of schistosomiasis in the field–even for low-intensity infections–in order to monitor the success of treatment and to evaluate control programs. This study was conducted to assess two tests frequently used regarding performance in comparison with real-time PCR. The routinely used KK thick smear assay is facile and highly specific. Nevertheless, it shows limited sensitivity in low intensity infections and it is unsuitable for assessment of the effect of mass drug administration on prevalence and infection intensity [21]. Our study was designed to provide better insight into the performance of POC-CCA test and schistosome-specific real-time PCR when on the one hand KK is used as the reference standard and on the other hand LCA is done. When using the KK method as reference to calculate the sensitivities and specificities of POC-CCA test and real-time PCR the sensitivities are high but the specificities are extremely low. These low specificities could be due to the low sensitivity of the Kato-Katz method. Using LCA, we were able to show that KK test has the lowest sensitivity compared to the other two methods. This analysis also showed very high sensitivities for POC-CCA and real-time PCR. A superior sensitivity of POC-CCA test in detecting *S*. *mansoni* infections in endemic areas has already been described in several other studies [19,38,40,41]. In their review Kittur *et al*. described that results obtained by POC-CCA and KK are similar, if KK prevalence is higher than 50%. If KK test prevalence is lower, the determined prevalence by POC-CCA assay can be much higher [42].It is known that some factors such as urinary tract infections, haematuria, use of diuretics etc. may alter the positivity of POC-CCA, particularly in weak positives [43]. This indicates that there might be a risk of overestimating the prevalence when using POC-CCA test, especially in low prevalence settings. In this study positive samples were overrepresented. The prevalence of *S*. *mansoni* has been between 85.2% and 94.9% depending on the method. Mugono *et al*. reported also that children in schools located near the lake shores have a higher infection rate than children located at a higher distance from the lake [8]. The determinant parameters to evaluate POC-CCA test depend on the prevalence of infection and test performance. Therefore, these aspects should be verified in low endemic settings.

In this study, *S*. *haematobium* infections were detected using a two-step test: only haematuria-positive urine samples were examined microscopically. Possible weak infections of *S*. *haematobium* could be overlooked, which could, however, have led to a positive result of POC-CCA test. When comparing the performance of POC-CCA assay to KK or PCR assays, *S*. *haematobium* infections should be known. On the other hand, several studies indicated that POC-CCA test is not reliable in detecting S. *haematobium* infections [16,42,44].

Another important finding of our study is the demonstrable decrease of sensitivity of POC-CCA test in individuals with low infection intensities (Fig 4). These results are comparable to other studies reported elsewhere [13,45,46]. The intensity of a positive POC-CCA test band reaction was strongly correlated with the egg count categories quantified by the KK method. The same results were also shown in previous studies [38,47]. Our present results also demonstrated that real-time PCR may be a good indicator for infection intensities as measured by a correlation between Ct-values and egg counts (Fig 3). The reason for this correlation might be that the DNA detected in faecal samples is most likely derived from eggs [48]. This outcome is contrary to that of Espírito-Santo *et al*. (2014), who did not find any correlation between Ct value and the number of eggs [49].

The sensitivity of real-time PCR was not higher than the sensitivity of POC-CCA test. In 3 samples, real-time PCR failed to detect *S*. *mansoni* infections, despite the microscopic finding of eggs. It could be possible that samples were swapped or mislabelled and urine and stool samples were not taken from the same participant. However, false negative results of PCR methods have been reported by different research groups [50,51]. Possible causes can be an inhibition of the amplification reaction by faecal compounds, degradation of DNA during transportation from the field or during storage of samples, variation in egg output and uneven distribution in faeces [51]. Interference of the amplification reaction due to inhibition could be excluded in our study because an internal control was used. Moreover, we used a real-time PCR set-up instead of a conventional PCR in order to avoid post-PCR handling and contamination.

The results of LCA demonstrated that real-time PCR and KK have the highest specificity. These findings are due to the fact that presents of eggs or *S*. *mansoni* specific DNA is highly specific. These findings are in contrast to a similar study performed by Al-Shehri *et al*. in 2018, where highest specificities have been obtained by KK and POC-CCA [37].

In conclusion, our results show that POC-CCA test is a valuable, easy-to-use and accurate test to detect *S*. *mansoni* infections in moderate or high endemic areas. In comparison to real-time PCR, there were only minor differences. Even in areas with low prevalence and intensity, the prevalence determined by POC-CCA is higher than that of KK [42]. Examinations of pre-school und school-aged children in a low prevalence area done by Okoyo *et al*. demonstrated a higher sensitivity of POC-CCA test in comparison with KK method. Referring to their results POC-CCA test is a sensitive and accurate screening tool for *S*. *mansoni* even in low prevalence areas [52]. Nevertheless, the KK test is often preferred in field surveys. It needs to be pointed out that the microscopic detection has major advantages: it is much more specific than POC-CCA test, a quantitative analysis is possible and at the same time other geohelminths can be detected [53].

From a scientific point of view, the method with the highest sensitivity and specificity should be used as diagnostic test. Although real-time PCR has a very good sensitivity and the highest specificity among the 3 tests used, this method is only partially suitable for application in the field. The biggest drawback is the required equipment. In addition, special trained personnel is necessary to carry out the test. Nevertheless, real-time PCR might be a useful additional tool to verify the standard methods or to detect light infections of *S*. *mansoni*.

## Supporting information

S1 Checklist STARD checklist. (PDF)

S1 Flowchart STARD diagram. (PDF)

S1 File CREC ethical clearance form. (PDF)

S2 File Informed consent. (PDF)

S3 File Dataset. (SAV)
