# Supplementary figures and images for: Comparison of sensitivity and specificity of three diagnostic tests to detect Schistosoma mansoni infections in school children in Mwanza region, Tanzania
Source: PLoS One. 2018 Aug 22;13(8):e0202499. doi: 10.1371/journal.pone.0202499 (PMC6105001; doi:10.1371/journal.pone.0202499)

STARD diagram to report flow of participants through the study

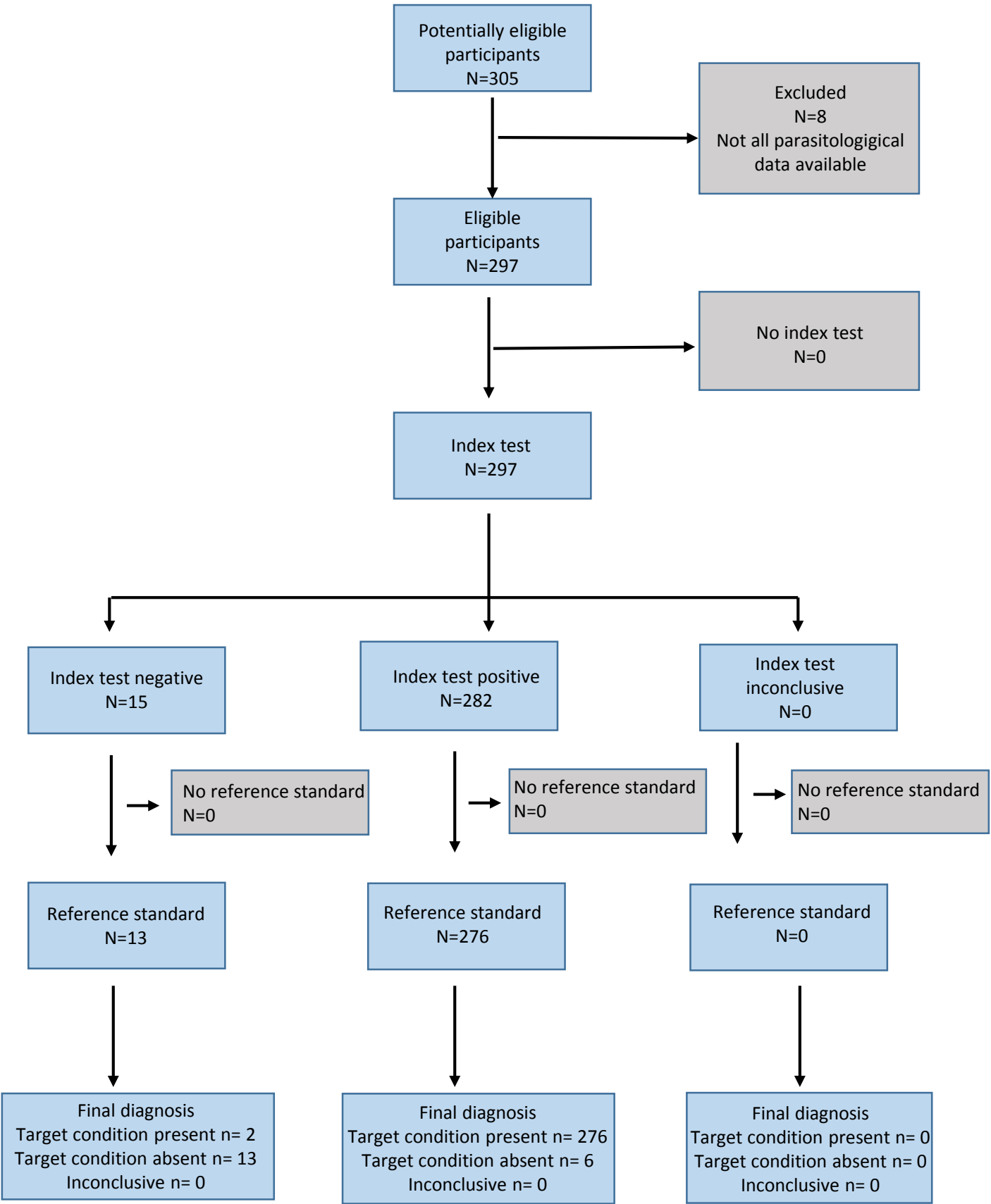

Supplement: S1 Flowchart — (PDF) [file pone.0202499.s002.pdf]
