# Supplementary material for: Comparison of sensitivity and specificity of three diagnostic tests to detect Schistosoma mansoni infections in school children in Mwanza region, Tanzania
Source: PLoS One. 2018 Aug 22;13(8):e0202499. doi: 10.1371/journal.pone.0202499 (PMC6105001; doi:10.1371/journal.pone.0202499)
Supplement: S1 File — (PDF) [file pone.0202499.s003.pdf]

CATHOLIC UNIVERSITY OF HEALTH AND ALLIED SCIENCES BUGANDO

P.O. Box 1464 Phone: (255) 28-250-0881 Email: principal@bugando.ac.tz

Mwanza, Tanzania Fax: (255) 28-250-2678 Website: www.bugando.ac.tz

CUHAS/BMC RESEARCH & ETHICAL COMMITTEE (CREC)
ETHICAL CLEARANCE FORM

**Date**	5 March 2015
**Research Clearance Certificate No**	**CREC/062/2014**
**Name of researcher/PI**	Antje Fuss
**Purpose of the research**	Pilot study
**Title of the Research**	Pilot study to evaluate the sensitivity of a modified technique of *Schistosoma* CCA detection in urine
**Budget and Sponsor(s)**	US$ 14,010/=<br>Wuerzburg Medical Mission Institute
**Research period**	March 2015 to August 2015

Ethical clearance is hereby granted.

A progress report shall be submitted to the Committee every 6 months.

Director RESEARCH AND PUBLICATION CATHOLIC UNIVERSITY OF HEALTH AND ALLIED SCIENCES - BUGANDO P.O. BOX 1464 MWANZA, TANZANIA.

CREC Chairperson

Director POSTGRADUATE STUDIES CATHOLIC UNIVERSITY OF HEALTH AND ALLIED SCIENCES - BUGANDO P.O. BOX 1464 MWANZA, TANZANIA.

CREC Secretary
