# Supplementary material for: Comparison of sensitivity and specificity of three diagnostic tests to detect Schistosoma mansoni infections in school children in Mwanza region, Tanzania
Source: PLoS One. 2018 Aug 22;13(8):e0202499. doi: 10.1371/journal.pone.0202499 (PMC6105001; doi:10.1371/journal.pone.0202499)
Supplement: S2 File — (PDF) [file pone.0202499.s004.pdf]

Appendix 1 – Informed consent in English
PARTICIPANT INFORMATION SHEET & CONSENT FORM

Study title

Pilot study to evaluate the sensitivity of a modified technique of *Schistosoma* CCA detection in urine

Invitation paragraph

You are being invited to take part in a research study. Before you decide it is important for you to understand why the research is being done and what it will involve. Please take time to read the following information carefully and discuss it with your parents / caretaker. Take time to decide whether or not you wish to take part.
Thank you for reading this.

Why is this study being done?

Schistosomiasis is a common parasitic disease in Tanzania. Schistosome infections may lead to reduced physical and cognitive development in children, decreased productivity of adults, organ damage, major morbidity, and in some cases, death. Early detection of parasite infestation and early treatment is important to prevent morbidity and mortality. The currently available tests are not sensitive enough to detect low infection levels.
In this study we want to evaluate the sensitivity of a modified technique of an already existing rapid diagnostic test.
If you / your child are / is diagnosed to be infected, he/she will be treated with the recommended drug Praziquantel.

Do I have to take part?

It is up to you to decide whether or not to take part. If you do decide to take part you will be given this information sheet to keep and be asked to sign a consent form together with your parent / caretaker. If you decide to take part you are still free to withdraw at any time and without giving a reason. A decision to withdraw at any time, or a decision not to take part, will not affect the standard of care you receive.

How many people will take part in the study?

About 300 children will take part in this study.

What will happen if I take part in this research study?

If you decide to take part in the study a morning urine sample and a stool specimen will be requested. No blood samples will be collected.

How long will I be in the study?

All of the procedures and tests will be done once only. It is not planned to do follow up investigations. Therefore you will not have any further obligations.

Can I stop being in the study?

Yes. You can decide at any time to withdraw your consent. The data obtained from you will be removed from the study data base and the specimen obtained will be destroyed.

What side effects or risks can I expect from being in the study?

To provide urine sample and stool specimen for laboratory investigations does not bear any risks for you. In case you are diagnosed to have a schistosoma infection you will be offered treatment with praziquantel. This is the WHO recommended drug of choice. It is the drug which is currently used in mass treatment campaigns and you may have received it already before. The side effects of a treatment with praziquantel are rare and usually mild. They may consist of abdominal or stomach discomfort with or without nausea for 1or 2 days.

Are there personal benefits to taking part in the study?

The laboratory investigations are looking for active Schistosomiasis, a worm disease that can reliably be treated with drugs. The rapid diagnostic tests will provide a result within a few days and you will receive a report as soon as possible. In case an active infection is found you will receive the appropriate medication free of charge. If you wish your study doctor will discuss the results of the laboratory tests with you. Some of the specialized tests will be done outside of Tanzania and the results may take a long time, e.g. several months. All results will be made available to you and your doctor as soon as possible.

What other choices do I have if I do not take part in this study?

Your physician will treat you according to your clinical needs and absolutely independent from your decision to take part in the study.

Will my medical information and my taking part in this study be kept confidential?

All information that is collected about you during the course of the research will be kept strictly confidential. Any information about you which leaves the hospital/surgery will have your name and address removed so that you cannot be recognized by a third party.

What are the costs of taking part in this study?

You will not need to pay for any study specific procedures.
You will not be paid for taking part in this study.

What happens if I am injured because I took part in this study?

As pointed out above it is very unlikely that you will be harmed or injured due to study specific procedures. But if you feel so, it is important that you tell your study doctor, ___________________ (*investigator's name).* You can tell the doctor in person or call him/her at ___________________ *[telephone number]*.

What are my rights if I take part in this study?

Taking part in this study is your choice. You may choose either to take part or not to take part in the study. If you decide to take part in this study, you may withdraw your consent at any time. In this case your data will be removed from the study database and your specimens destroyed.

No matter what decision you make, there will be no penalty for you and you will not lose any of your regular benefits. Withdrawing your consent will not affect your medical care. You can still get your medical care from our institution.

Who can answer my questions about the study?

You can talk to your study doctor about any questions or concerns you have about this study. Contact your study doctor __________________ *[name]* at __________________ *[telephone number]*.

Additional Information about your rights while taking part in this study, can be obtained by the Institutional Review Board of the Catholic University of Health and Allied Sciences (CUHAS).

Please give your consent

Signature

I have been given a copy of all 3 pages of this form. I have read it or it has been read to me. I understand the information and have had my questions answered.

I agree to take part in this study. ☐ YES

Date ____

Signature of participant: ____

Signature of legal representative: ____

(parent / caretaker)
